# Seamless assembly of DNA parts into functional devices and higher order multi-device systems

**DOI:** 10.1371/journal.pone.0199653

**Published:** 2019-06-28

**Authors:** Jeffrey Carl Braman, Peter J. Sheffield

**Affiliations:** Agilent Technologies, Inc., La Jolla, CA, United States of America; Imperial College London, UNITED KINGDOM

## Abstract

A new method is introduced allowing seamless assembly of independent, functionally tested, blunt-end double strand nucleic acid *parts* (DNA fragments not supplied in vectors such as plasmids) into more complex biological *devices* (e.g. protein expression vectors) and higher order multi-device *systems* (e.g. biochemical pathways). Individual *parts* include bacterial selection markers and origins of replication, promoters useful in a variety of species, transcription terminators, shuttle sequences and a variety of “N” and “C” terminal solubility/affinity protein tags. *Parts* are not subjected to pre-assembly manipulation with nucleic acid modifying enzymes. Instead, they are simply mixed in appropriate pre-defined combinations and concentrations and then seamlessly linked into *devices* employing a specialized thermostable enzyme blend. Combinatorial assembly of *parts* is an inherent time-saving feature of the new method, in contrast to hierarchical binary assembly (“one part at a time”) methods. This feature substantially simplifies and speeds optimization of *device* and *system* development. The versatility and functionality of the new method was shown by combinatorial assembly of *parts* into vector *devices*, one of which optimally expressed protein from a model gene. Also, a four-enzyme biosynthetic pathway *system* was re-created by combinatorial construction from *parts and devices*. Concepts discussed in this paper provide synthetic biologists, chemists and bio-engineers with improved and expanded capability to create novel biological molecules and *systems*.

## Introduction

The discipline of synthetic biology has greatly benefitted from key enabling technologies such as DNA synthesis and sequencing becoming accessible to more researchers due to the reduction in the previously prohibitive financial entry point. To date however, a third enabling technology, molecular cloning, has not kept pace with technological advances made in DNA synthesis and DNA sequencing. One of the most highly recognized collection of techniques and materials developed to improve conventional cloning of biological *parts*, *devices* and *systems* is **“**BioBricks**”** [1, 2]. Briefly, the “bricks”, or *parts*, of this technology represent cloned DNA sequences possessing defined functions, such as antibiotic resistance and ribosome binding sites. *Parts* are assembled to create larger *devices* such as protein expression vectors and several *devices* are joined into a *system* such as a biosynthetic pathway. BioBrick *devices* and *systems* are constructed by “hierarchical binary assembly” of *parts*, or “one-brick-at-a-time.” More specifically, BioBricks represent functional double strand DNA molecules housed within carrier plasmids flanked by universal and precisely defined upstream and downstream sequences that are technically not part of the BioBrick. These universal sequences contain restriction enzyme recognition sites for one of two closely related enzymes, each having slightly different recognition sequences but upon cleavage generate identical termini (isocaudomers). Linking two BioBricks together requires isolation of the individual *parts* from their carrier plasmids by specific isocaudomer(s) digestion, end repair in some cases, ligation and finally bacterial transformation. A major drawback to this technique is that BioBrick *parts* must not contain these restriction enzyme recognition sites within the sequences to be assembled. Also, BioBrick hierarchical binary assembly is time consuming, tedious and not conducive to combinatorial assembly.

Current assembly methods that convert *parts* into *devices* also rely on the isolation of *parts* and *devices* from dedicated BioBrick-like “destination vectors” (BioBricks [1, 2], SLIC [3], Gibson [4], CPEC [5], SLiCE [6], and In-Fusion [http://www.clontech.com/US/Products/Cloning_and_Competent_Cells/Cloning_Resources/Selec-tion_Guides/In-Fusion_Cloning_Kits]). In other methods, significant *parts* manipulation with either one or more Type-II restriction enzymes is required (GoldenGate [7], MoClo [8], GoldenBraid [9]). Alternatively, *parts* manipulation with T5-exonuclease or a combination of Pfu and Taq DNA polymerases are required for Gibson [4] and DATEL [10] assembly methods, respectively, to create overlaps for subsequent annealing and ligation. In summary, assembly methods are complicated when restriction enzyme specificity must be considered at each stage of *parts* and *devices* design. Also, creating small *parts* between 50 and 250 base pairs with one or more enzymes possessing exonuclease activity is difficult due to the propensity of these enzymes to completely degrade the *parts*. It is apparent that these limitations curtail combinatorial experimental design and significantly slow the process of identifying optimal *devices* and *systems*.

Providing functionally validated *parts* to researchers without the need for retrieval from destination vectors, combined with a seamless protocol conducive to combinatorial assembly of *parts* into *devices* and higher order *systems* would represent a significant improvement in synthetic biology molecular cloning. This paper describes such a system. *Parts* are provided such that a wide variety of *devices* can be rapidly assembled. Appropriately chosen *parts* are combined and assembled in a single-tube reaction creating molecules that transform/transfect and properly function as *devices* in *E*. *coli*, mammalian and yeast (*Saccharomyces cerevisiae*) cells. Multiple assemblies can be performed in parallel to generate a collection of unique *devices* that can be used in combination to optimize novel biological *systems*. The utility of this technology, referred to as “SureVector” (SV), was validated by assembling *parts* into a collection of plasmid *devices* that were easily screened to identify clones expressing the largest amount of protein from a model gene of interest (GOI). To further demonstrate the applicability of the SV process, a *multi-device system* was designed and constructed to reconstitute a four-enzyme biosynthetic pathway in *E*. *coli*. This *multi-device system* enabled identification of several over-expressing clones within one week.

## Materials and methods

### SureVector (SV) p*arts*

SureVector *parts* include bacterial origins of replication [OR], bacterial selectable markers [SM], XP-1 and XP-2 referred to as “Expansion *parts*” containing sequences allowing replication and selection in a variety of organisms (*Saccharomyces cerevisiae* and mammalian cells; defined in more detail below), promoters [P] (*E*. *coli*, *Saccharomyces cerevisiae* and mammalian), and protein expression tags [T] (both C-terminal and N-terminal tags are available). Each *part* is flanked by unique 30 base pair (bp) sequences not found in any global DNA databases, such as NCBI’s Basic Local Alignment Search Tool BLAST (http://blast.ncbi.nlm.nih.gov/Blast.cgi), and allow specific assembly of *parts* into *devices* (example shown in Table 1) and ultimately *systems*.

**Table 1:** SureVector overlaps (N-terminal fusions).

5' overlap		Fragment	3' overlap	
OV 6	GCTCATTTCTACAACGCGGCACTTCTCGAG	Promoter	CCTTGTTTAACTTTAAGAAGGAGATATACAT	G4S
G4S	CCTTGTTTAACTTTAAGAAGGAGATATACAT	Expression Tag	GGTGGCGGAGGTTCTGGAGGCGGTGGAAGT	OV 5
OV 5	GGTGGCGGAGGTTCTGGAGGCGGTGGAAGT	GOI 5’ → 3’	CATTTGGTTTAGTGTACAATATCTCCTCGAG	OV 4
OV 4	CATTTGGTTTAGTGTACAATATCTCCTCGAG	Expansion Slot 2	CAACAGGAGGTGAAGCTGTAACGTCTCGAG	OV 3
OV 3	CAACAGGAGGTGAAGCTGTAACGTCTCGAG	Expansion Slot 1	GATTTTGAGACGTGCTCACAGTTTTCTCGAG	OV 2
OV 2	GATTTTGAGACGTGCTCACAGTTTTCTCGAG	Origin of Replication	CAGTTCTTCTGGTTGGAGGACTTCCTCGAG	OV 1
OV 1	CAGTTCTTCTGGTTGGAGGACTTCCTCGAG	Bacterial Selectable Marker	GCTCATTTCTACAACGCGGCACTTCTCGAG	OV 6

### XP1 and XP2 expansion *parts*

XP1 *parts* contain either the yeast autonomous replication sequence (γARS or 2-micron circle) allowing plasmid replication in *Saccharomyces cerevisiae* or a linker derived from a unique nucleotide sequence not possessing a function other than to tether a chosen bacterial origin of replication to an XP2 fragments during *parts* assembly.

The XP2 *parts* include either a non-functional tether sequence, as above, or the lacI repressor found in *E*. *coli*, or mammalian selection markers or yeast auxotrophic markers, allowing yeast to grow in the absence of an essential amino acid. XP2 *parts* designed specifically for use in mammalian cells include blasticidin, puromycin or hygromycin selection markers. For yeast, XP2 *parts* include the auxotrophic markers URA3 and HIS3 and the selection marker for hygromycin resistance. XP2 *parts* also contain a transcription terminator; either the bovine growth hormone poly-A signal (bGH pA) specific for mammalian cells or a rho independent terminator for bacterial transcripts provided by the strong hairpin forming sequence (5’-GCCGCCAGCGGAACTGGCGGC-3’). These terminator sequences are positioned and oriented within the XP2 *part* to terminate transcripts from an upstream GOI.

### Large scale production and purification of SV *parts*

Large scale *parts* synthesis was performed by PCR in 96-well plates using sequence verified master plasmid templates. Each reaction contained 1 ng of plasmid template, 1x Herculase II reaction buffer (Agilent Technologies, Inc.), 0.25 mM of each dNTP, 0.4 μM of each PCR primer and 2 μl of pre-formulated Herculase II enzyme (Agilent Technologies, Inc.) in a final volume of 100 μL. Thermocycling conditions were: 1 cycle at 95°C for 2 min.; 30 cycles at 95°C for 20 sec., 55°C for 20 sec. and 72°C for 30 sec.; 1 cycle at 72°C for 3 min. Contents of multiple 96-well plates for each SV *part* were pooled and purified using AMPure XP magnetic beads according to the manufacturer’s instructions (Beckman-Coulter). Correct lengths and purities of SV *parts* were assessed with an Agilent Technologies, Inc. BioAnalyzer.

### General assembly of SV *parts* into *devices*

SV *parts* designed to assemble into a desired *device* were combined with a SV adapted GOI *part* and other reaction components were added as follows: 1x SureVector reaction buffer, 0.25 mM of each dNTP, 5.0 nM of each *part* (e.g. SM + OR + XP1 + XP2 + T + GOI + P) and 1 μL of pre-formulated thermostable enzymes including high fidelity DNA polymerase, dUTPase, “flap-endonuclease” and ligase in a final volume of 20 μL. Thermocycling of these components consisted of 1 cycle at 95°C for 2 min.; 8 cycles at 95°C for 20 sec., 55°C for 20 sec. and 68°C for 30 sec.; 1 cycle at 68°C for 3 min. Following thermocycling, one unit of *Dpn* I restriction enzyme was added to the reaction and incubated for 5 minutes at 37°C. One μL of the reaction was transformed into XL1-Blue Supercompetent *E*. *coli* cells (Agilent Technologies, Inc.) according to instructions and varying amounts (10, 20 and 50 μL) of the transformation mixtures were spread onto LB agar plates containing the appropriate antibiotic and incubated at 37°C until colonies were easily visualized (12–16 hrs.). *Device* DNA was purified from select colonies and either analyzed by restriction digestion, or sequenced to verify correct *parts* assembly, or used directly in downstream processes.

### SV *parts* assembly into Nedd5 protein expression *devices*

To demonstrate an obvious use of the new SV cloning method, the human Nedd5 gene was chosen as a model GOI for performing an expression screening experiment. Nedd5 is a mammalian septin known to associate with actin-based structures such as the contractile ring and stress fibers and is involved in the process of cytokinesis in human brain tumors [11], although the specific nature of Nedd5 is not pertinent to this paper. The Nedd5 gene containing a start and a stop codon was adapted by PCR to be SV compatible by using the primers listed below:

N-terminal forward primer 5’- GGTGGCGGAGGTTCTGGAGGCGGTGGAAGT***ATG***GGATCCATGTCTAAGCAACAACCAACTC-3’ and N-terminal reverse primer 5’- TCGAGGAGATATTGTACACTAAACCAAATG***TCA***CACATGCTGCCCGAGAGCCCCGCTGTCAC-3’.

The Nedd5 gene containing a start codon but lacking a stop codon was adapted by PCR to be SV compatible by using the following primers:

C-terminal forward primer 5’- CCTTGTTTAAACTTTAAGAGGAGGGCCACC***ATG***GGATCCATGTCTAAGCAACAACCAACTC-3’ and C-terminal reverse primer 5’-CCACCGCCTCCAGAACCTCCGCCACCCACATGCTGCCCGAGAGCCCCGCTGTCACTGTCAC-3’.

Primers show the start codon or stop codon in bold-italicized type and all primers include unique 30 bases (underlined) for assembly with adjacent *parts*. The resulting Nedd5 PCR products were used as the model GOI and assembled into a variety of twelve expression constructs each containing a different C- or N-terminal expression tag. The following *parts* were used in these assemblies: Amp [SM] + pBR322 [OR] + XP-1 linker + XP-2 *lacI* + Nedd5 [GOI] with either C-terminal Tags[T] (c-Myc, thioredoxin, streptavidin binding protein, calmodulin binding protein, His6 or hemagglutinin) or N-terminal tags (GST, MBP, His6, SBP, CBP, or HisDbsA, calmodulin binding protein or hemagglutinin) + pTac [P] (Refer to section entitled “General assembly of SV *parts* into *devices*”). As a comparison the Nedd5-Fusion expression region from these SureVector assembled plasmids was isolated by PCR and sub-cloned, using restriction enzymes, into a commercially available ptac based expression vector. Both plasmids were then subjected to the identical protein expression protocol.

### SV Nedd5 expression *devices* screening

SureVector derived, and the ptac constructed, Nedd5 clones with different expression tags were cultured overnight at 37°C with shaking at 250 rpm in 1 ml of LB broth containing ampicillin (50 μg/ml). The following day, 10 ml cultures of LB broth containing ampicillin (50 μg/ml) were inoculated with these clones and incubated at 37°C with shaking at 250 rpm until the OD_600,_ a measure of bacterial growth, reached 0.6 (time zero, approximately 1 hour). Protein expression was induced by the addition of IPTG to a final concentration of 0.5 mM followed by incubation for 20 hours at 30°C with shaking at 250 rpm. Volumes of cells equal to OD_600_ of 3.0 were removed from each culture at time zero (uninduced samples) and after 20 hours of incubation (induced samples). The samples were then centrifuged, and cell pellets resuspended in 120 μl of 8M urea. The mixtures were vortexed well and incubated at 75°C for 5 min. Cell lysates were centrifuged and supernatants analyzed for Nedd5 expression by SDS-gel electrophoresis.

### SV assembly of *parts* and *devices* into higher order *systems e*xpressing DMRL

SV *parts* and *devices* were assembled to reconstitute the four enzyme *E*. *coli* biosynthetic pathway (*system*) for 6,7-dimethy-8-ribityllumazine (DMRL), the fluorescent precursor to riboflavin (rib pathway genes). The DMRL *system* construction was accomplished by first assembling *devices* with zero, one or two rib genes. PCR primers used to amplify rib open reading frame gene *parts* A (ribA– 591 bp), B (ribB– 654 bp), D (ribD– 1104 bp) and E (ribE—471 bp) with appended SV overlaps to make them SV compatible were:

*E*. *coli* ribA–Gene ID = 945763

1. ribA_Forward Primer-N-Tag (56 bp)

ggtggcggaggttctggaggcggtggaagt***ATG***CAGCTTAAACGTGTGGCAGAAGC

2. ribA_Reverse Primer-RBS (67 bp)

gaaattgttaaattatttctagattcgaaaggagctcgaattcTTATTTGTTCAGCAAATGGCCCAT

*E*. *coli* ribB–Gene ID = 947526

1. ribB_Forward Primer-N-Tag (56 bp)

ggtggcggaggttctggaggcggtggaagt***ATG***AATCAGACGCTACTTTCCTCTTT

2. ribB_Reverse Primer-RBS (68 bp)

gaaattgttaaattatttctagattcgaaaggagctcgaattcTCAGCTGGCTTTACGCTCATGTGCC

*E*. *coli* ribD–Gene ID = 945620

1. ribD_Forward Primer-RBS (73 bp)

ttcgaatctagaaataatttaacaatttcacataaaggaggtaaata***ATG***CAGGACGAGTATTACATGGCGCG

2. ribD_Reverse Primer (54 bp)

ctcgaggagatattgtacactaaaccaaatgTCATGCACCCACTAAATGCAGGC

*E*. *coli* ribE—Gene ID = 946453

1. ribE_Forward Primer-RBS (74 bp)

ttcgaatctagaaataatttaacaatttcacataaaggaggtaaata***ATG***AACATTATTGAAGCTAACGTTGC

2. ribE_Reverse Primer: (55 bp)

ctcgaggagatattgtacactaaaccaaatgTCAGGCCTTGATGGCTTTCAATAC

Two sets of bi-cistronic *devices* were designed and assembled, one containing the ribA and ribD genes and the other containing the ribB and ribE genes. A ribosome binding site (RBS) was included in the 3’ region of the ribA and ribB genes downstream of their native stop codon and the same sequence was also included in the 5’ region upstream of the ATG start codon of the ribD and ribE genes. This RBS sequence was used as the overlap by which the ribD and ribE genes were positioned downstream of ribA and ribB genes, respectively. The intended outcome was to place two rib genes under control of one promoter and couple expression of the upstream and downstream rib genes via a second RBS between the two rib genes. This was done to attempt to balance expression levels of the individual rib genes. This second RBS overlap region between the rib genes was designed in such a way that the downstream rib gene was not in the same reading frame as the upstream rib gene thus preventing two gene products in the same *device* from becoming physically linked. An additional stop codon was also added to each upstream rib gene to further guard against translation read-through. Bi-cistronic vectors of this type have been used previously for preparation of platelet activating factor acetylhydrolase (PAF-AH) alpha domain heterodimer for crystal structure studies [12] and for the analysis of BORG/Septin heterodimer filament formation [13]. *Devices* lacking either the upstream or downstream rib gene *parts* were correctly assembled into circular molecules using 90 bp N-terminal and C-terminal “Non-Coding” linker *parts* NC-N and NC-C, respectively, and were made by overlap extension [14]:

N-terminal “NC” replaces ribA or ribB *parts*

rib—“NC” N-term_Forward ggtggcggaggttctggaggcggtggaagtgaaactgcactcatcgtccctcgaggagct

rib—“NC” N-term_Reverse gaaattgttaaattatttctagattcgaagagctcctcgagggacgatgagtgcagtttc

C-terminal “NC” replaces ribD or ribE *parts*

rib—“NC” C-term_Forward ttcgaatctagaaataatttaacaatttcacataaaggaggtatagacagcatacgagtc

rib—“NC” C-term_Reverse ctcgaggagatattgtacactaaaccaaatgactcgtatgctgtctatacctcctttatg

Bi-cistronic *devices* that only contain a single rib gene required either a NC-N or NC-C *part* in lieu of the corresponding rib gene *part*. Three standard *parts* were used in both sets of rib *devices*; the T7 promoter-HIS6, XP1 linker and XP2 lacI. These 3 standard *parts* were used in various combinations with either the ampicillin or kanamycin selectable markers and the pBR322 or p15a bacterial origins of replication. Therefore, all SV rib *devices* were assembled from just seven SV *parts*. A total of 18 *device* level plasmids were constructed. These system level *devices* were designated by letter-number codes. “K” *devices* consisted of the kanamycin resistance marker (kan), the p15a origin of replication and either zero, one or two rib genes. “A” *devices* consisted of the ampicillin resistance marker (amp), the pBR322 origin of replication and either zero, one or two rib genes (Table 2). Higher order *systems* were created using various combinations of these *device* level plasmids by the co-transformation of two *devices*. For example, *devices* K6 and A7 resulted in co-expression of ribA-ribD genes from the K6 *device* and ribB-ribE genes from the A7 *device*. Combinations of *devices* were transformed into Agilent BL21(Gold) DE3 *E*. *coli* and spread onto LB-agar plates containing 100 μg/ml each of kanamycin and ampicillin (LB-kan-amp) plus 0.5 mM IPTG. Plates were incubated at 37°C for 12 to 18 hours and examined under UV light to identify DMRL expressing clones (*systems*) as evidenced by fluorescent colonies surrounded by fluorescent halos.

**Table 2:** SV *devices* required to re-create the DMRL biosynthetic pathway.

**Kanamycin/p15a Rib “K” *Devices***						
***Device* ID**	**SM**	**Origin**	**Promoter**	**GOI1**	**GOI2**	**Type**
K1	Kan	p15a	T7-His	RibA	NC-C	Control
K2	Kan	p15a	T7-His	RibB	NC-C	Control
K3	Kan	p15a	T7-His	NC-N	RibD	Control
K4	Kan	p15a	T7-His	NC-N	RibE	Control
K5	Kan	p15a	T7-His	NC-N	NC-C	Control
K6	Kan	p15a	T7-His	RibA	RibD	Test Plasmids
K7	Kan	p15a	T7-His	RibB	RibE	Test Plasmids
K8	Kan	p15a	T7-His	RibA	RibE	Test Plasmids
K9	Kan	p15a	T7-His	RibB	RibD	Test Plasmids
**Ampicillin/pBR322 Rib “A” *Devices***						
***Device* ID**	**SM**	**Origin**	**Promoter**	**GOI1**	**GOI2**	**Type**
A1	Amp	pBR322	T7-His	RibA	NC-C	Control
A2	Amp	pBR322	T7-His	RibB	NC-C	Control
A3	Amp	pBR322	T7-His	NC-N	RibD	Control
A4	Amp	pBR322	T7-His	NC-N	RibE	Control
A5	Amp	pBR322	T7-His	NC-N	NC-C	Control
A6	Amp	pBR322	T7-His	RibA	RibD	Test Plasmids
A7	Amp	pBR322	T7-His	RibB	RibE	Test Plasmids
A8	Amp	pBR322	T7-His	RibA	RibE	Test Plasmids
A9	Amp	pBR322	T7-His	RibB	RibD	Test Plasmids

Each *device* contains zero, one or two rib genes, and either the kanamycin resistance marker and p15a origin of replication prefabs (labeled “K”) or the ampicillin resistance marker and pBR322 origin prefabs (labeled “A”). Single rib gene control plasmids (K1 –K4; A1-A4) require either N- or C- terminal “non-coding” *parts* (NC-N and NC-C, respectively) to assemble complete *devices*. Zero rib gene control *devices* (K5 and A5) contain both NC-N and NC-C *parts* in place of both rib genes to assemble into complete *devices*. Co-transformation of one “K” *device* (e.g. K6 through K9) with one “A” *device* (e.g. A6 through A9) results in a higher order *system* expressing either 0, 1, 2, 3 or 4 rib genes.

### Validation of DMRL *system* synthesis—assay, purification and MS analysis

Monitoring clones for DMRL synthesis on agar plates was straightforward as the DMRL fluoresces within colonies and is secreted into the surrounding media creating fluorescent halos around DMRL positive colonies. DMRL production from clones cultured in liquid media was also straightforward as the compound possesses a characteristic visible light absorption spectrum with λ max OD_490_ [15] that can be measured in cell free supernatants. Validation of DMRL synthesis required its production, purification and analytical characterization. Clone K6A7 produced pronounced fluorescent colony-halos and was chosen for this purpose. A single colony was inoculated into 3 ml of LB-kan-amp liquid media and incubated overnight at 37°C with shaking at 250 rpm. A 2.0 ml sample of this culture was inoculated into a 500 ml Erlenmeyer flask containing 100 ml of LB-kan-amp and incubation continued as before until the OD_600_ value reached 0.35. IPTG was added to a final concentration of 0.5 mM and incubation continued for an additional 18 hours. Cells were removed by centrifugation and acetic acid added to the supernatant to a final concentration of 5%. This sample was applied to a 2.5 x 3.5 cm column of Florisil (Sigma-Aldrich) equilibrated with 5% acetic acid. The column was washed with one liter of 5% acetic acid and DMRL eluted with 100 ml of 3% pyridine. Solvent was removed by evaporation and the residue suspended in 5 ml of water. This sample was applied to a 2.5 x 35 cm column of chromatography grade cellulose (Sigma-Aldrich) equilibrated with water and DMRL was eluted with water. Forty milligrams of DMRL were recovered from 0.8g of cells (wet weight) and was analyzed by mass spectrometry. The sample was run on an Agilent 6538 QTOF coupled to an Agilent 1100/1200 lc stack. The column was a Zorbax SB-C18 0.5 x 150 mm. Flowrate = 20μl/min. Injection volume = 5μl. Solvent A was H_2_O – 0.1% formic acid and solvent B was acetonitrile– 0.1% formic acid. Gradient: T0: 95% A—5% B; T10: 75% A—25% B; T12: 50% A—50% B; T15: 5% A—95% B; T20: off. Five min. re-equilibration time. For the ms run, a range from m/z = 85 to m/z = 1100 was scanned. For the ms/ms run, m/z 327.1 was targeted, with three different collision energies: ce = 10V, ce = 20V, and ce = 40V.

### Quantities and rates of DMRL synthesis from *devices and systems*

Single colonies from *devices* and *systems* listed in Table 2 were purified by re-streaking onto fresh LB-kan-amp plates without IPTG. Three colonies from each plate were cultured overnight at 37°C with shaking at 250 rpm in separate tubes containing 3 ml of LB-kan-amp. The next day, one hundred microliters of each culture were added to 4.9 ml of LB-kan-amp and incubated until OD_600_ reached between 0.3 and 0.5. IPTG was added to a final concentration 0.5 mM and incubation continued for three hours after which OD_600_ values were re-measured. Cultures were centrifuged to remove cells and OD_409_ values of supernatants obtained. OD_409_ values were normalized relative to OD_600_ values measured post IPTG addition and the resulting numbers compared. DMRL was synthesized exclusively by clones containing all four rib genes (two devices each expressing two rib genes)–*systems* K6A7, K7A6, K8A9, K9A8.

DMRL synthesis rates of *systems* K6A7, K7A6, K8A9, K9A8 and negative control K5A5 were obtained by inoculating single colonies into 5 ml of LB-kan-amp media followed by overnight incubation at 37°C with shaking at 250 rpm. One ml of these starter cultures was inoculated into separate 250 ml flasks each containing 49 ml of LB-kan-amp. Incubation at 37°C with shaking at 250 rpm continued until OD_600_ values reached between 0.3 and 0.5. IPTG was added to a final concentration of 0.5 mM and incubation continued. At regular intervals, 1.0 ml samples were retrieved from each culture and OD_600_ values measured. Samples were then centrifuged to remove cells and OD_409_ values of supernatants obtained. OD_409_ values were normalized relative to OD_600_ values measured post IPTG addition and the resulting numbers compared.

## Results and discussion

### Design features of SV *parts* and assembly process

**Fig 1:**
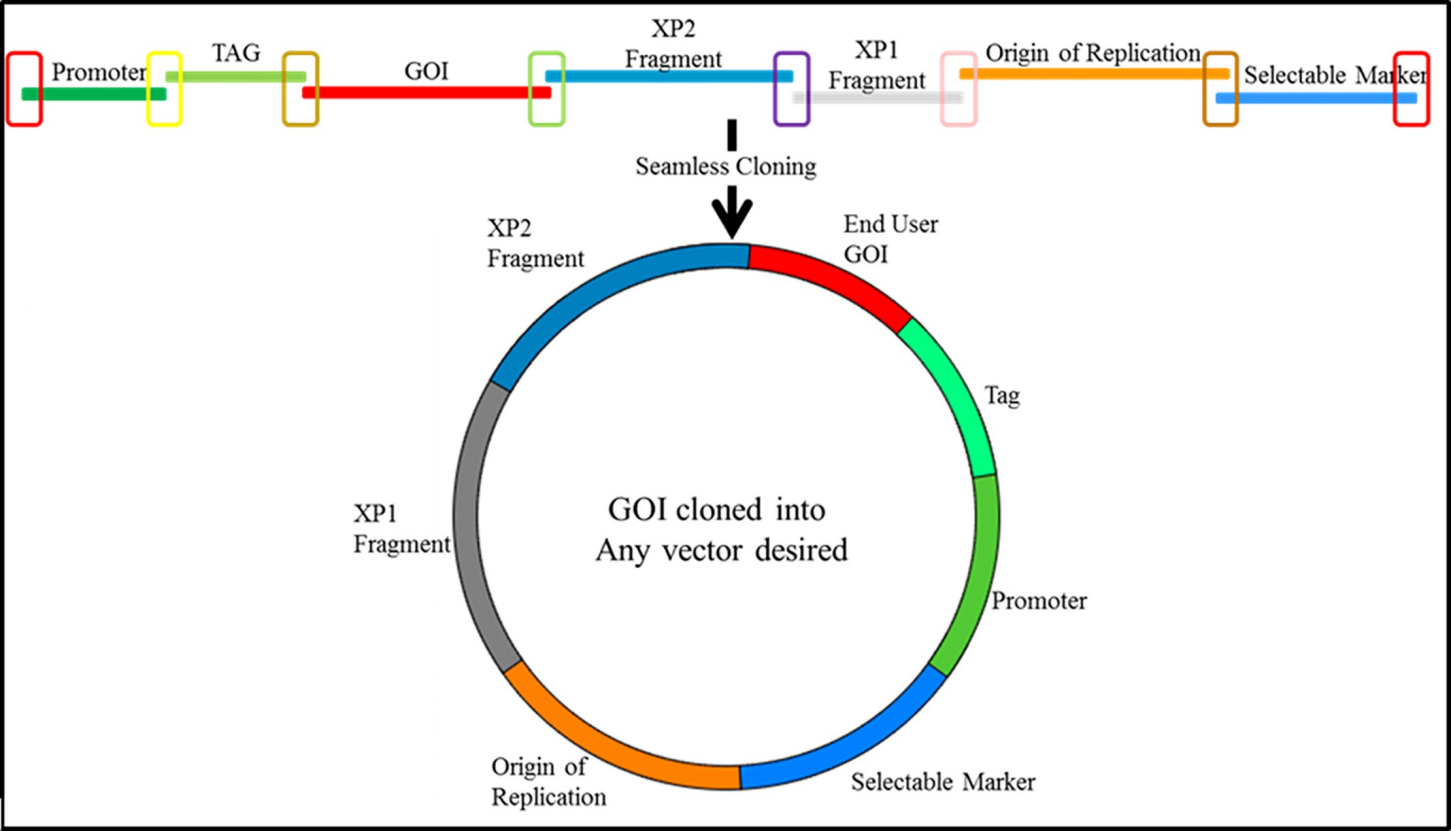
Schematic of a seven SV *part* assembly into a GOI expressing *device*. Different colored open rectangles highlight unique 30 bp overlaps between functional *parts*. Sequences represented by the two end rectangles (red) also overlap. The mixture of *parts* is treated with the SureVector enzyme assembly blend resulting in a *device*, represented by the closed circle that will transform, replicate and express a GOI in *E*.*coli*.

The key design features of SV *parts*, ensuring precise and ordered joining, are the unique 30 bp sequences incorporated into each PCR primer used to generate a *part* (see Materials and Methods section). Fig 1 schematically represents how seven SV *parts* align and overlap due to this design feature.

Assembling *parts* listed in Table 1 in the manner described in the Materials and Methods section and in the Fig 1 legend generated *devices* expressing GOI’s with an N-terminal fusion protein: -Bacterial Selectable Marker-Bacterial Origin of Replication-XP1-XP2-GOI←Expression Tag←Promoter- (← denotes direction of promoter and gene expression)

**Fig 2:**
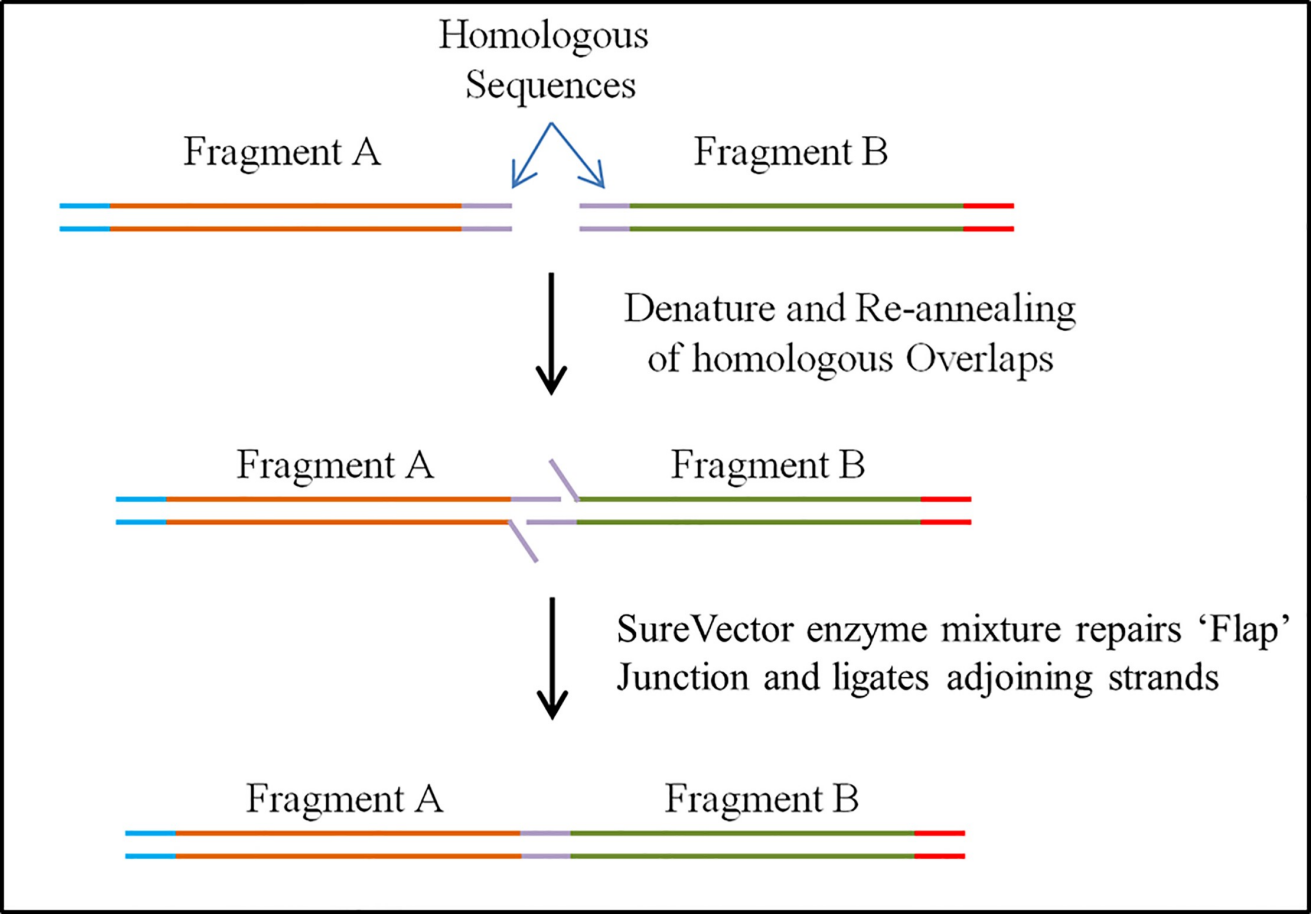
Schematic Showing How Adjacent SV P*arts* are Assembled; *Parts* A and B Possess Homologous Ends. Following denaturation and annealing, resulting free 3’ ends are extended, “flaps” digested and the two parts ligated.

The assembly mechanism of linking *parts* into higher order *devices* is represented in Fig 2. *Parts* are denatured, and adjacent *parts* anneal due to the 30 bp overlaps. Exposed 3’-OH ends are partially extended by a polymerase resulting flaps that are digested by an endonuclease and covalently joined by a ligase.

**Fig 3:**
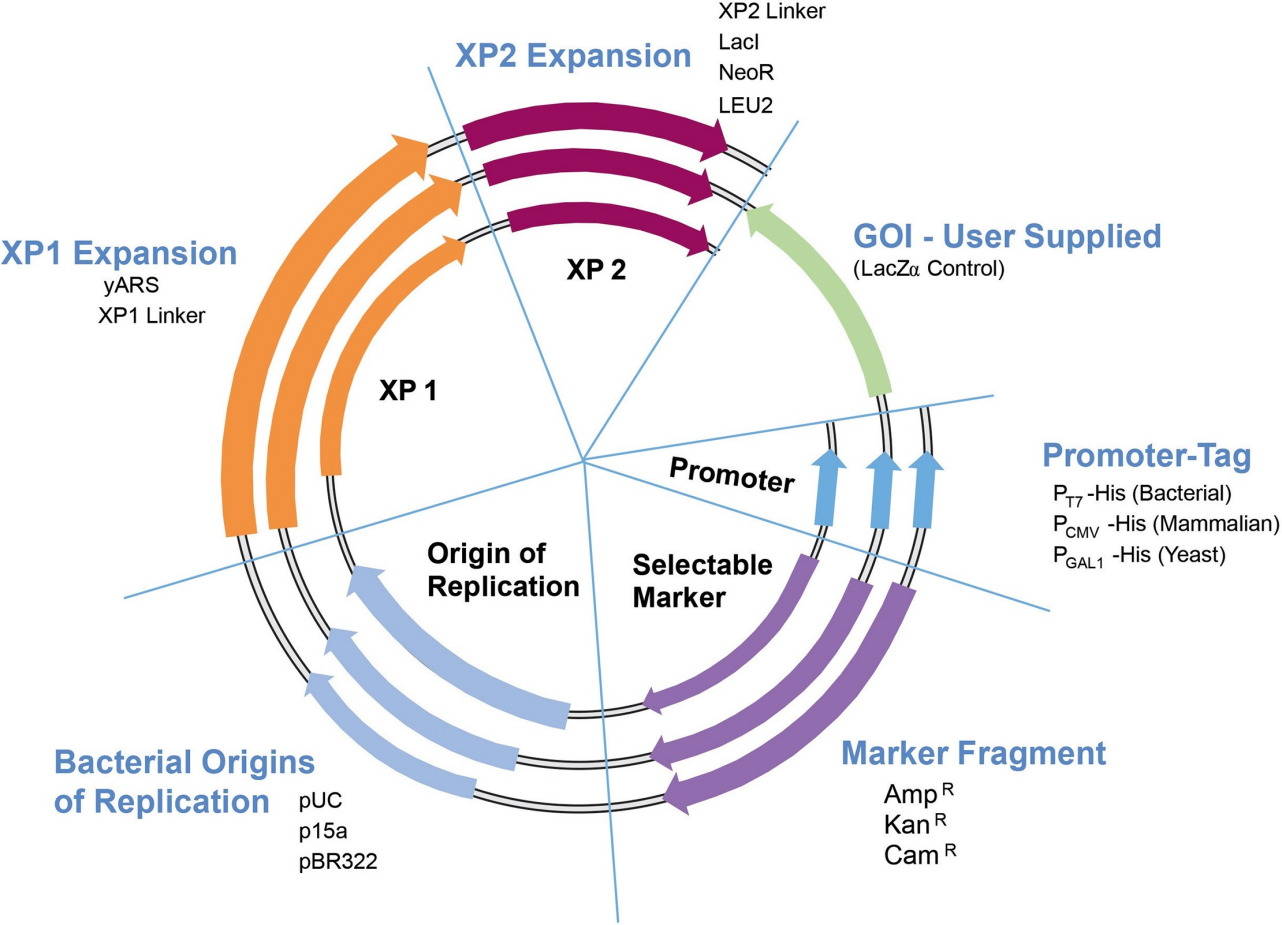
Collection of SV *parts* and assembly design. A variety of *parts* and GOI’s can be assembled into many functional *devices*.

Fig 3 represents the combinatorial assembly power of the SV process. Different functional *parts* are rapidly assembled into multiple configurations in parallel assembly experiments to determine the best organization for, in this case, expression of a single GOI.

### SV Nedd5 expression *devices* screening

**Fig 4:**
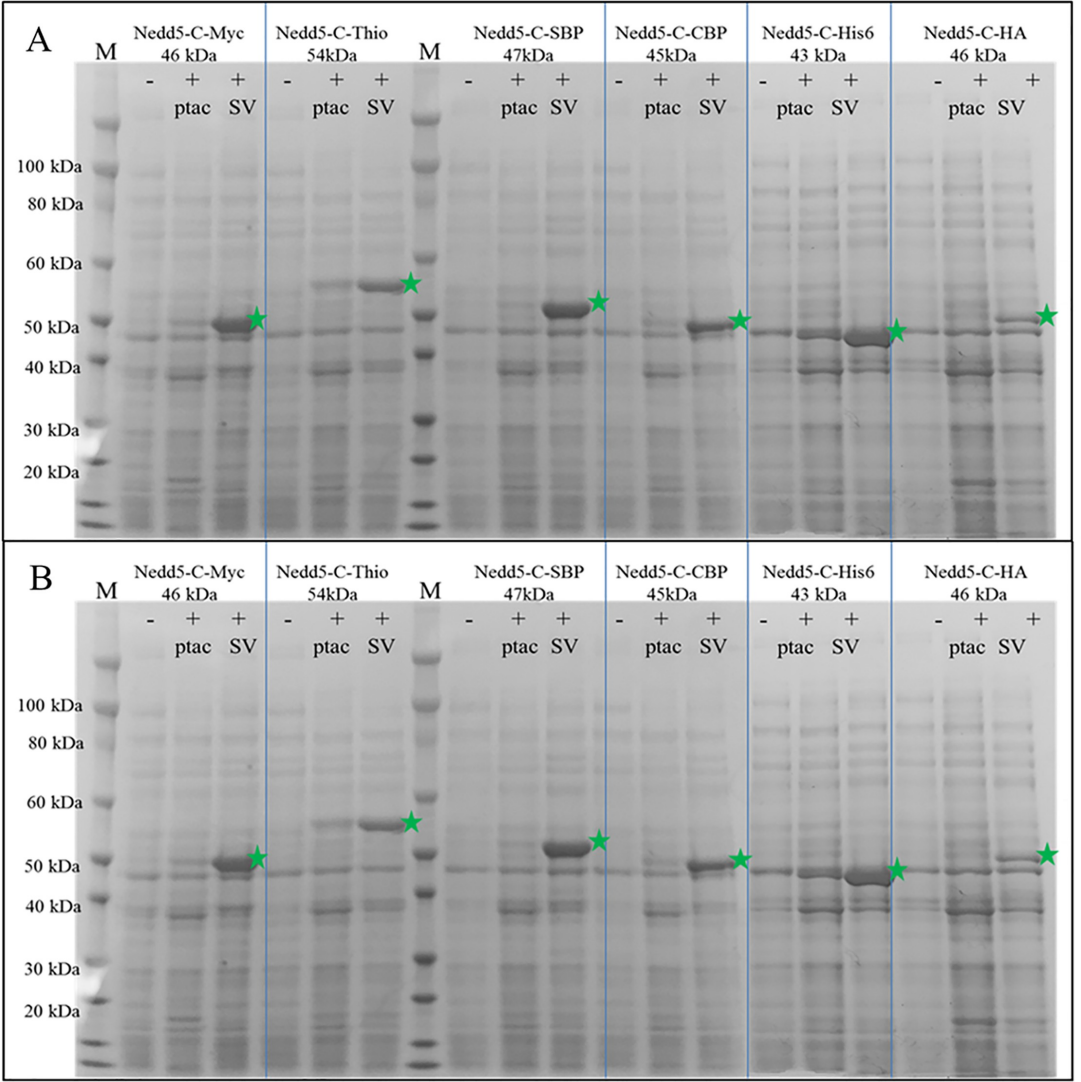
**a. SDS-PAGE gel of SV Nedd5 expression *devices* with different N-terminal tags.** M = protein molecular weight marker; (-) = Uninduced sample; (+) Induced samples. SV denotes expression from SureVector assembled plasmids, ptac denotes expression from the commercially available ptac based plasmids. Green stars denote expressed N-terminal tagged Nedd5-fusion proteins. **Fig 4b. SDS-PAGE Gel of SV Nedd5 Expression *Devices* with Different C-Terminal Tags.** M = protein molecular weight marker; (-) = Uninduced samples; (+) = Induced samples. SV denotes expression from SureVector assembled plasmids, ptac denotes expression from the commercially available ptac based plasmids. Green stars denote expressed N-terminal tagged Nedd5-fusion proteins.

SDS-page analysis of twelve Nedd5 expression *devices*, fused with N- or C- terminal tags identified the best expression constructs (green arrows shown in Fig 4A and Fig 4B highlight proteins from induced cultures). Quantities of expressed fusion proteins varied with the type of expression tag and were highest with N-terminal tagged MBP/Nedd5, HisDbsA/Nedd5, and CBP/Nedd5 *devices* and C-terminal tagged Nedd5/SBP, Nedd5/c-myc and Nedd5/His6 *devices*. Lesser quantities of fused proteins were expressed as N-terminal tagged GST/Nedd5 and His6/Nedd5 *devices* and C-terminal tagged Nedd5/Thioredoxin and Nedd5/CBP *devices*. The least quantities of fusion Nedd5 were N-terminal tagged SBP/Nedd5 and C-terminal tagged Nedd5/HA *devices*. It is worth emphasizing that this protein expression screening experiment, starting from assembly of *parts* into *devices* and analyzing protein expression was completed in less than three days.

### Re-creating a biosynthetic pathway

**Fig 5:**
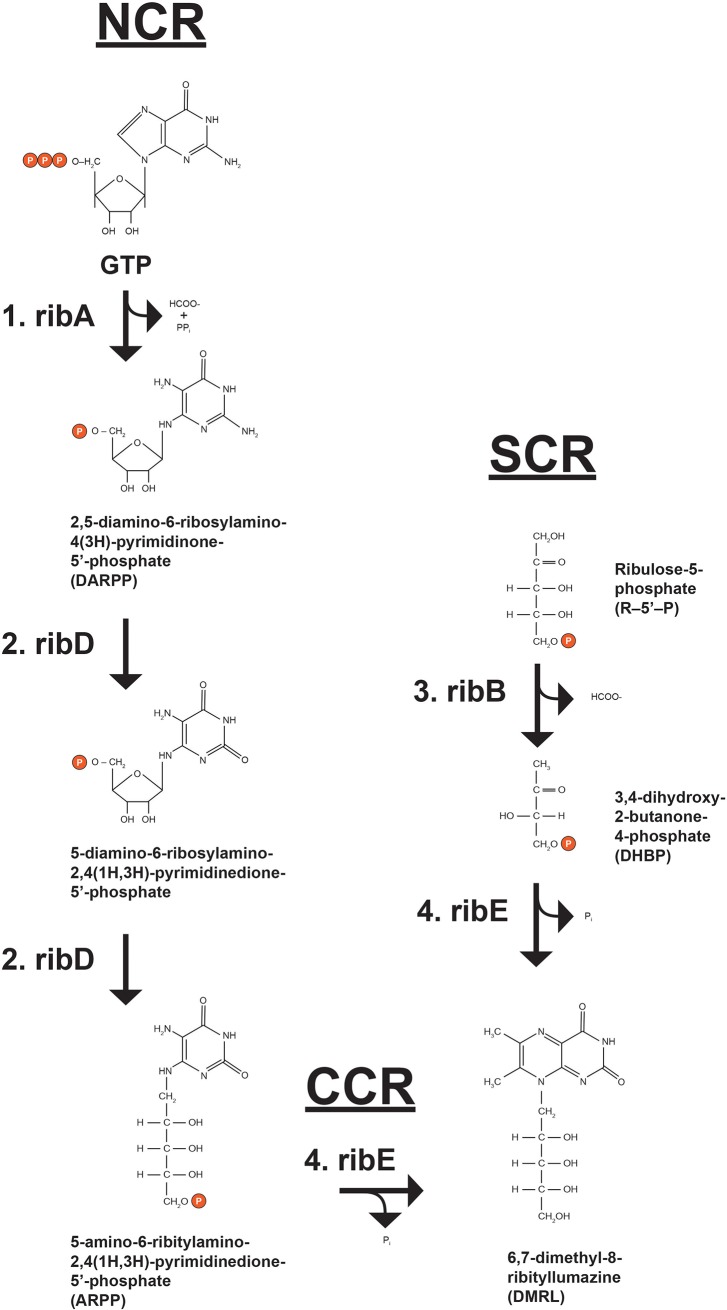
DMRL biosynthetic pathway. The imidazole ring of GTP is hydrolytically removed by GTP Cyclohydrolase II (rxn. 1; ribA) yielding 2,5-diamino-6-ribosylamino-4(3H)-pyrimidinone-5’-phosphate (DARPP), formate and pyrophosphate. DARPP is converted to 5-amino-6-ribitylamino-2,4(1H,3H)-pyrimidinedione-5’-phosphate (ARPP) by fused diaminohydroxyphosphoribosylaminopyrimidine deaminase/5-amino-6-ribitylamino-2,4(1H,3H)-pyrimidinedione reductase (rxns. 2; both ribD). Separately, ribulose-5-phosphate (R5P) is converted to L-3,4-dihydroxy-2-butanone-4-phosphate (DHBP) and formate by L-3,4-dihydroxy-2-butanone-4-phosphate synthase (rxn. 3; ribB). ARPP and DHPB are dephosphorylated and then condensed by DMRL synthase (rxn. 4; ribE) producing 6,7-dimethyl-8-ribityllumazine (DMRL).

The utility of SV *parts* assembly into *devices* and ultimately higher order *systems* was demonstrated by recreating the *E*. *coli* biosynthetic pathway for 6,7-dimethy-8-ribityllumazine (DMRL), the fluorescent precursor to riboflavin (Fig 5; [16]). DMRL is synthesized by four unique enzymes (expressed from ribA, ribB, ridD and ribE genes) and substrates. The critical initial substrates for this pathway are GTP and ribulose-5’-phosphate (R5P). They are funneled through a “nucleotide conversion route” (NCR), a “sugar conversion route” (SCR) and a “converging condensation route” (CCR). The NCR starts by the hydrolytic removal of carbon atom 8 from the imidazole ring of GTP by GTP Cyclohydrolase II (ribA gene product; [17]) yielding 2,5-diamino-6-ribosylamino-4(3H)-pyrimidinone-5’-phosphate (DARPP). DARPP is converted to 5-amino-6-ribitylamino-2,4(1H,3H)-pyrimidinedione-5’-phosphate (ARPP) by diaminohydroxyphosphoribosylaminopyrimidine deaminase/5-amino-6-ribitylamino-2,4(1H,3H)-pyrimidinedione reductase (ribD gene product; [18, 19]), a “fused” enzyme possessing both NCR and SCR-relevant activities. As the enzyme name describes, the nucleotide base of DARPP is deaminated followed by reduction of the ribosyl moiety to ribityl with NADPH serving as reductant. Separately, L-3,4-dihydroxy-2-butanone-4-phosphate synthase, an enzyme exclusively in the SCR (ribB gene product; [20]) converts ribulose-5’-phosphate (R-5-P) to L-3,4-dihydroxy-2-butanone-4-phosphate (DHBP) and formate. Both ARPP and DHBP are dephosphorylated and enter the CCR via DMRL synthase (ribE gene product; [21]) resulting in DMRL.

### Pathway construction

DMRL pathway construction was accomplished by first assembling SV *parts* into *devices* (Table 2) containing zero, one and two (“bi-cistronic”) rib genes. PCR primers used to amplify rib gene *parts* A, B, D and E are shown in Materials and Methods. They allowed amplification of rib open reading frames ribA– 591 bp, ribB– 654 bp, ribD– 1104 bp and ribE—471 bp with appended correct overlaps to make them SV compatible. Two sets of bi-cistronic *devices*, one containing the ribA and ribD genes and the other containing the ribB and ribE genes were also designed and assembled (Table 2). Ribosome binding sites (RBS) were designed and incorporated into the 3’and 5’ flanking regions of the rib genes and subsequently used as unique overlaps between the genes such that ribD and ribE genes were positioned downstream of the ribA and ribB genes, respectively. The intended outcome of placing two rib genes under control of one T7 promoter and coupling expression of the upstream and downstream rib genes was balanced expression levels. The RBS placed in the 5’ regions of both ribD and ribE genes promoted downstream rib gene translation efficiency. The RBS overlap between the upstream and downstream rib genes was designed so that the downstream rib genes were out of frame with the upstream rib genes thus preventing two gene products in the same *device* from becoming physically linked. An additional stop codon was also added to each upstream rib gene as an added prevention to translation read-through. As mentioned previously, bi-cistronic vectors of this type have been used previously for preparation of platelet activating factor acetylhydrolase (PAF-AH) alpha domain heterodimer for crystal structure studies [12] and for the analysis of BORG/Septin heterodimer filament formation [13]. SV *devices* lacking one or both rib genes were also assembled using N-terminal and C-terminal “Non-Coding” *parts*; NC-N and NC-C, respectively (Table 2). Zero or single rib gene control *devices* required either an NC-N or NC-C *part*, or both in the case of the double negative control, in lieu of a rib gene *part*. Common SV *parts* used in both sets of rib *devices* were the T7 promoter-HIS6, XP1 linker and XP2 lacI (allowing IPTG induction of rib gene *devices* expression) in addition to various combinations of ampicillin and kanamycin selectable markers with pBR322 or p15a bacterial origins of replication. Therefore, all 18 SV rib *device* plasmids were assembled from just 7 seven SV *parts*, not including the rib gene *parts*.

**Fig 6:**
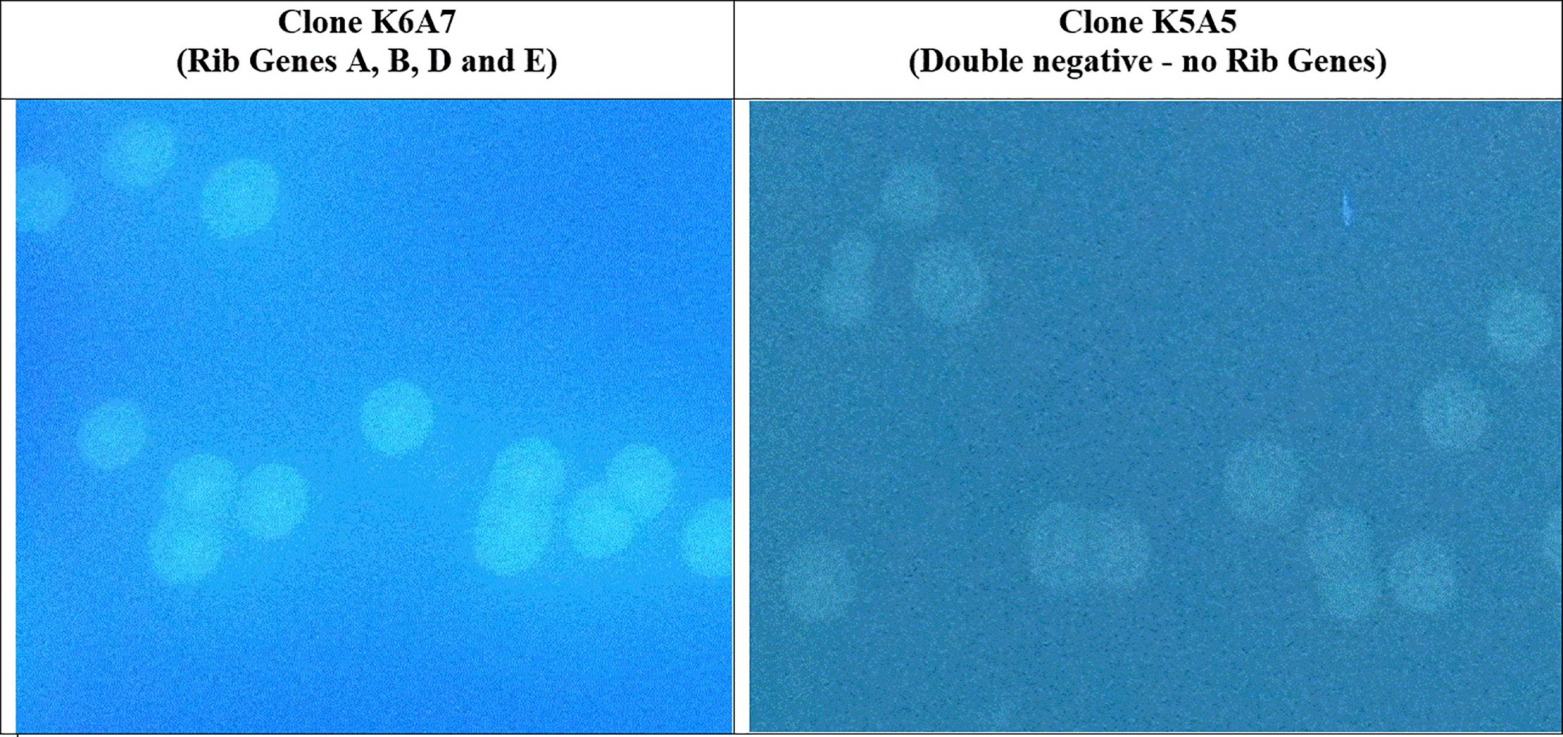
Co-transformation of two SV compatible *devices* containing all 4 rib biosynthetic genes results in DMRL synthesis. *Devices* K6 and A7 (see Table 2) were co-transformed into Agilent BL21(Gold)DE3 *E*.*coli* and spread onto LB-kan-amp-IPTG plates. Resulting colonies were examined under unfiltered UV light. All resulting K6A7 *systems* contained four rib genes and produced DMRL as evidenced by fluorescent colonies with fluorescent halos. Control (*device* K5A5) through three rib genes (see Table 2) did not produce DMRL as no fluorescent halos were detected.

Table 2 *devices* were transformed either individually or in various co-transformation scenarios (Table 3) into Agilent BL21(Gold) DE3 *E*. *coli* and spread onto LB- agar plates containing 100 μg/ml each of kanamycin and ampicillin (LB-kan-amp) plus 0.5 mM IPTG. Plates were incubated at 37°C for twelve to eighteen hours. All colony *devices* and *systems* were easily monitored for DMRL synthesis by irradiating plates with UV light and visualizing fluorescent colonies surrounded by fluorescent halos. DMRL producing colony *systems* contained all four rib genes (Fig 6 - left panel). Colonies containing only 1, 2 or 3 rib genes, as well as the double negative control lacking all 4 rib genes (clone K5A5 assembled with NC-N and NC-C *parts*), did not produce DMRL (Fig 6 - right panel; no fluorescent colonies and no halos).

**Table 3:** Combinations of Table 2 functional and control *devices* used to re-create the DMRL biosynthetic pathway *system*.

Transformations into BL21(GOLD)					
*System*		rib Genes Expressed	Rib genes Missing	Plate on	Assay
*Device* 1	*Device* 2				
K1	-	A	B, D and E	Kan	Control
K2	-	B	A, D and E	Kan	Control
K3	-	D	A, B and E	Kan	Control
K4	-	E	A, B and D	Kan	Control
K5	-	-	A, B, D and E	Kan	Control
K6	-	A and D	B and E	Kan	Control
K7	-	B and E	A and D	Kan	Control
K8	-	A and E	B and D	Kan	Control
K9	-	B and D	A and E	Kan	Control
A1	-	A	B, D and E	Amp	Control
A2	-	B	A, D and E	Amp	Control
A3	-	D	A, B and E	Amp	Control
A4	-	E	A, B and D	Amp	Control
A6	-	A and D	B and E	Amp	Control
A7	-	B and E	A and D	Amp	Control
A8	-	A and E	B and D	Amp	Control
A9	-	B and D	A and E	Amp	Control
A5	-	-	A, B, D and E	Amp	Control
K1	A9	A, B and D	E	Kan+Amp	Control
K2	A8	B, A and E	D	Kan+Amp	Control
K3	A8	D, A and E	B	Kan+Amp	Control
K4	A9	E, B and D	A	Kan+Amp	Control
K6	A7	A, D, B and E	-	Kan+Amp	Test
K7	A6	B, E, A and D	-	Kan+Amp	Test
K8	A9	A, E, B and D	-	Kan+Amp	Test
K9	A8	B, D, A and E	-	Kan+Amp	Test

### Characterization of DMRL synthesized by SV rib *system* K6A7

DMRL synthesized and purified from *system* K6A7 (Table 3) was characterized by mass spectrometry (see Materials and Methods). For the ms run, a range from m/z = 85 to m/z = 1100 was scanned. The theoretical m/z value for DMRL is 327.12990 and the observed m/z value was 327.12914. In addition, for the ms/ms run the m/z value of 327.1 was targeted, using three different collision energies at 10V, 20V, and 40V. The expected exact mass of fragmented DMRL was 192.06 and the observed value was 192.07 confirming that SV *system* K6A7 produced DMRL.

### Quantities and rates of DMRL synthesis from DMRL *devices and systems*

**Fig 7:**
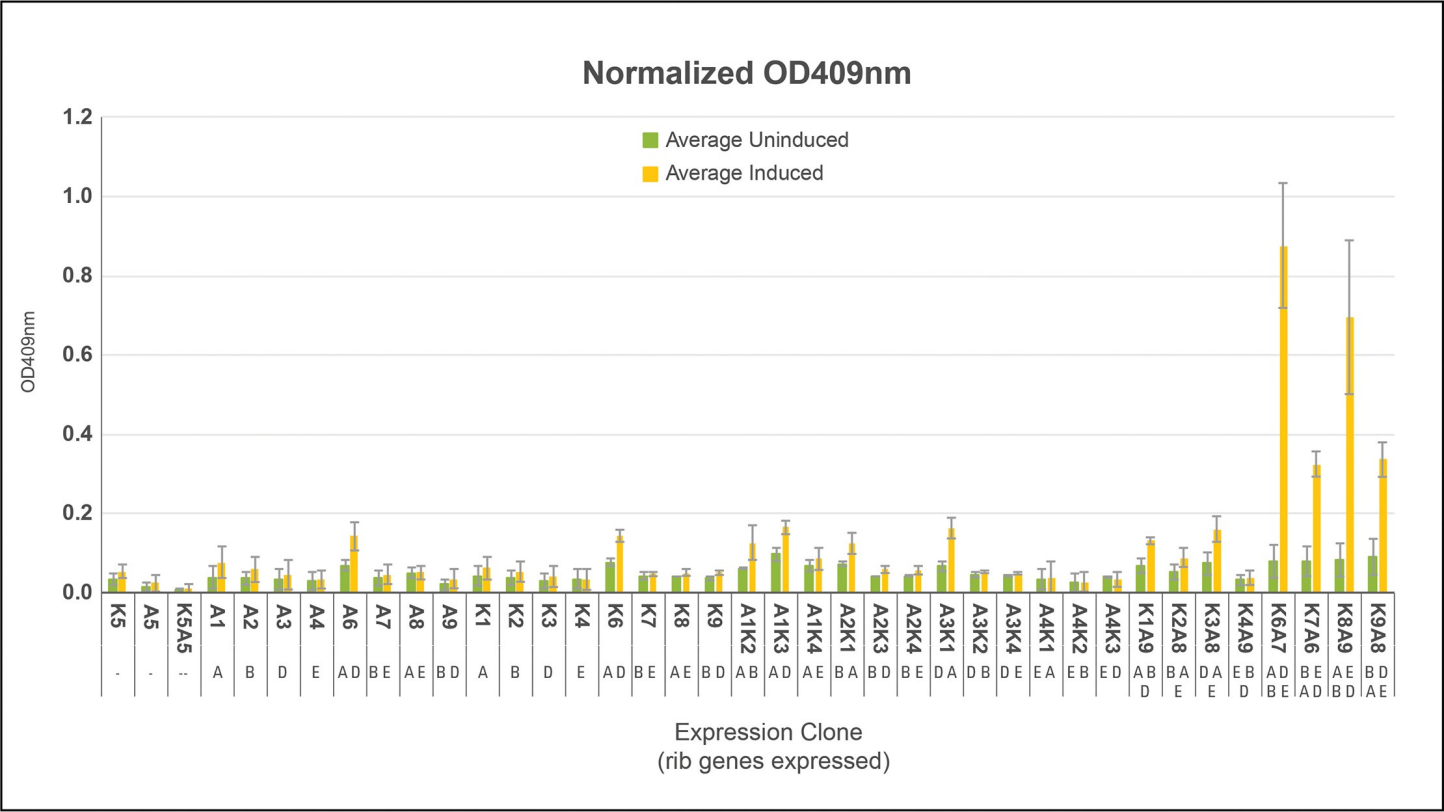
Total quantities of DMRL synthesis from *devices* and *systems*. Single colonies from *devices* and *systems* listed in Table 3 were purified by re-streaking onto fresh LB-kan-amp plates without IPTG. Three colonies from each plate were cultured in separate tubes containing 3 ml of LB-kan-amp. One hundred microliters of each culture were added to 4.9 ml of LB-kan-amp and incubated until OD_600_ reached between 0.3 and 0.5. IPTG was added to a final concentration 0.5 mM and incubation continued for three hours after which OD_600_ values were re-measured. Cultures were centrifuged to remove cells and OD_409_ values of supernatants obtained. OD_409_ values were normalized relative to OD_600_ values measured post IPTG addition and the resulting numbers compared. DMRL was synthesized exclusively by *systems* containing two *devices* each expressing two rib genes–*systems* K6A7, K7A6, K8A9 and K9A8.

**Fig 8:**
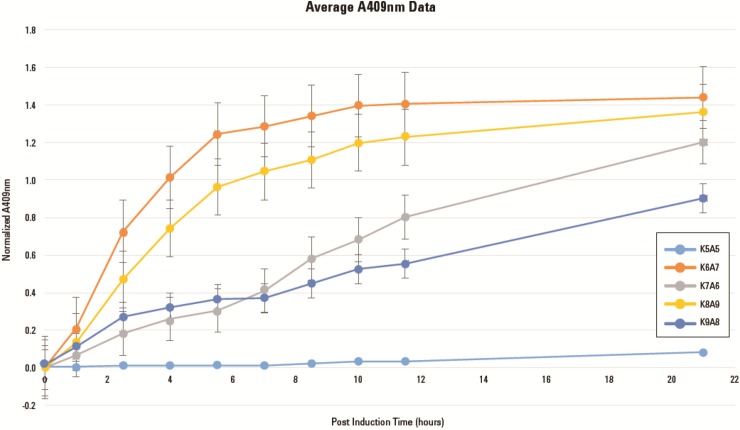
DMRL synthesis rates. DMRL synthesis rates of *systems* K6A7, K7A6, K8A9, K9A8 and negative control K5A5 were obtained by inoculating single colonies into 5 ml of LB-kan-amp media followed by overnight incubation at 37°C with shaking at 250 rpm. One ml of these starter cultures was inoculated into separate 250 ml flasks each containing 49 ml of LB-kan-amp. Incubation at 37°C with shaking at 250 rpm continued until OD_600_ values reached between 0.3 and 0.5. IPTG was added to a final concentration of 0.5 mM and incubation continued. At regular intervals, 1.0 ml samples were retrieved from each culture and OD_600_ values measured. Samples were then centrifuged to remove cells and OD_409_ values of supernatants obtained. OD_409_ values were normalized by dividing by the OD_600_ values and these numbers plotted as a function of OD_409_ post-IPTG addition.

Fig 7 confirms that only SV *systems* K6A7, K7A6, K8A9, K9A8, each containing two rib gene *devices* expressing all four rib genes, produced DMRL. *Systems* K6A7 and K8A9 produced the highest levels of DMRL and most rapidly (Fig 8). Both *systems* are composed of d*evices* K6 and K8 expressing enzymes from ribA and ribD genes and from ribA and ribE genes, respectively. The ribA gene is positioned immediately downstream of the T7 promoter in both K6 and K8 *devices*. In *devices* A7 and A9, the ribB gene is downstream of the T7 promotor. Total DMRL accumulation and slower synthesis rates are characteristic of *systems* K7A6 and K9A8. These *systems* are composed of d*evices* K7 and K9 expressing enzymes from ribB and ribE genes and from ribB and ribD genes, respectively. In K7 and K9 *devices* the ribB gene is positioned immediately downstream of the T7 promotor. The A6 and A8 *devices* express enzymes from ribA and ribD genes and ribA and ribE genes, respectively. In these *devices* ribA is downstream of the T7 promoter. How these *system* configurations resulted in different totals and rates of DMRL synthesis were not examined. However, there may be a connection between over-expression of ribA (GTP Cyclohydrolase II, the first committed step in the NCR) and production of DARPP (Fig 5) in kanamycin resistant hosts that leads to saturating substrate levels for the balance of NCR and CCR enzymes. Also, over-expression of ribB (L-3,4-dihydroxy-2-butanone-4-phosphate synthase, the first committed step in the SCR) and production of DHBP in ampicillin resistant hosts leads to saturating co-substrate levels for the CCR enzyme DMRL synthase (Fig 5). The net result of attaining saturating levels these substrates would be that all DMRL synthesis enzymes could operate at V_max_.

## Conclusions

A new method (SureVector) for seamless assembly of biological *parts* into functional *devices* and higher order *systems* is presented that takes advantage of principles learned from prefabrication engineering design and assembly. These are: (1) Functional DNA *parts* are manufactured and quality controlled at a location away from the assembly site (Agilent Technologies, Inc.); (2) *Parts*, other requisite assembly materials and detailed assembly instructions are delivered to the site of construction (the research laboratory) for assembly; (3) from a synthetic biology standpoint, processes (1) and (2) enable rapid and reliable combinatorial assembly of desired *devices* destined for introduction into *E*. *coli*, mammalian and yeast cells. The salient features of this new method were demonstrated by constructing a set of protein expression *devices* to identify the best *device* for the expression of a target GOI (Nedd5). To further illustrate the adaptability of this assembly method, a higher order plasmid *system* was constructed to recreate a four-gene biosynthetic pathway. The biosynthetic pathway to synthesize DMRL was selected as it served as a good model of a multi-gene system that could be easily evaluated. The combinatorial assembly power of this process was also utilized in developing a synthetic DMRL expression *system*. A total of 18 protein expression *devices* were assembled in one day possessing zero, one or two rib gene (bi-cistronic) *parts*. Expression screening was initiated the following day and positive *systems* were selected for structural and functional testing in less than one week. While assembled *devices* were being sequenced for validation of their structural integrity, DMRL was purified and characterized by mass spectrometry. Experiments were also performed to determine optimal DMRL synthesis rates and maximum production. The same type of project out-sourced to a third-party vendor would have taken several months to complete (previous experience). While optimizing DMRL production was not the goal of these experiments, it is worth noting that 40 mg of DMRL was purified from 100 ml of media collected after growth of 0.8 g (wet weight) of *E*. *coli* clone K6A7. In stark contrast, Maley and Plaut (15) required 5 kg of the mold *A*. *gossypii* to obtain 160 mg of DMRL.

The combinatorial power, simplicity and assembly accuracy of the SureVector process will facilitate building many “multi-device” *systems* including unique biochemical synthetic pathways and novel regulatory circuits. Production of fine chemical intermediates and end-products represents an obvious high-value application.
